# Age‐Related Trajectories of Autistic Traits in Children With Angelman Syndrome

**DOI:** 10.1002/aur.70017

**Published:** 2025-03-21

**Authors:** Doesjka A. Hagenaar, Sabine E. Mous, Leontine W. ten Hoopen, André B. Rietman, Kamil R. Hiralal, Karen G. C. B. Bindels‐de Heus, Pieter F. A. de Nijs, Theresa C. Mohr, Eline J. Lens, Manon H. J. Hillegers, Henriette A. Moll, Marie‐Claire Y. de Wit, Gwen C. Dieleman

**Affiliations:** ^1^ ENCORE Expertise Centre for Neurodevelopmental Disorders Erasmus MC Rotterdam the Netherlands; ^2^ Department of Child‐ and Adolescent Psychiatry/Psychology Erasmus MC Rotterdam the Netherlands; ^3^ Department of Pediatrics Erasmus MC Rotterdam the Netherlands; ^4^ Department of Neurology and Pediatric Neurology Erasmus MC Rotterdam the Netherlands

**Keywords:** Angelman syndrome, Autism Spectrum Disorder, autistic traits, longitudinal, repeated measures, sensory processing

## Abstract

Angelman syndrome (AS) is a rare neurogenetic disorder. Previous studies indicate a high prevalence of autism spectrum disorder (ASD) with considerable variability. Little is known regarding the longitudinal trajectory of autistic traits. We aim to investigate autistic traits, the effect of age on these traits, and associated features in AS children. This (partly) longitudinal clinical record study at the ENCORE Expertise Center involved 107 AS children aged 2–18 with one (*N* = 107), two (*N* = 49), or three (*N* = 14) measurements. Autistic traits and sensory processing issues were assessed using various instruments, and DSM classifications were used descriptively. Covariates were genotype, gender, and epilepsy. Results indicate a high prevalence of autistic traits and sensory processing issues. Children with the deletion genotype exhibited more autistic traits. Autism Diagnostic Observation Schedule (ADOS) classifications indicated higher rates of ASD compared to clinician DSM classifications. Autistic traits generally remained stable over time, except that ADOS scores significantly decreased for children with the *UBE3A* mutation genotype, and in the social affect domain for the entire group. In conclusion, incorporating the assessment of autistic traits and sensory processing into clinical practice for AS is important to inform adaptations of the environment to meet the child’s needs. Additionally, clinicians and researchers should be mindful of the potential for overestimating ASD traits in AS when relying on the ADOS. ASD diagnosis in AS should integrate multiple diagnostic instruments, diverse hetero‐anamnestic sources, and multidisciplinary expert opinions.

**Summary**

- Angelman syndrome (AS) is a rare genetic disorder often linked with autism.
- We found that autistic traits are common and generally stable over time as children with AS age, with some improvement in social interactions for those with a UBE3A mutation.
- It is important for clinicians and researchers to consider both autistic traits and sensory processing when working with children with Angelman syndrome. This could help create a better environment that meets their needs. However, they should also be cautious when using a common autism assessment tool (the ADOS), as it may overestimate autism traits in these children.

## 1 Introduction

Angelman syndrome (AS) is a rare neurodevelopmental disorder with an estimated birth incidence of 1 in 24.500–40.000 (Mertz et al. 2013; Thomson et al. 2006). It is caused by a loss‐of function of the *UBE3A* gene on the maternal chromosome 15q11–q13, which can be due to a deletion (60%–70%), a pathogenic variant of the *UBE3A* gene (*UBE3A* mutation; 15%), a paternal uniparental disomy (pUPD; 5%–10%), or an imprinting center defect (ICD; 5%–10%) (Lossie et al. 2001). Individuals with the deletion genotype generally have a more severe phenotype, while UPD and ICD genotypes are generally less affected and are phenotypically more alike (Lossie et al. 2001). Phenotypical characteristics of AS include severe intellectual disability (ID), speech impairment, motor or balance disorders, as well as a high prevalence of epilepsy and sleeping problems (Williams et al. 2006). Behavioral issues such as hyperactivity/impulsivity, anxiety, and autistic traits often occur (Wink et al. 2015; Wheeler et al. 2019).

Autism Spectrum Disorder (ASD) is characterized by deficits in social communication/interaction, restricted and repetitive behaviors, and sensory processing issues. Symptoms of ASD are present from early development and result in significant impairments in daily life functioning (Lord et al. 2018). A review of evidence indicates that the severity of autistic traits may change over time as individuals age, with the most common pattern being a decrease over time (Waizbard‐Bartov and Miller 2023; Pender et al. 2020). However, decreases in autistic traits over time have consistently been associated with higher IQ and the absence of ID (Waizbard‐Bartov and Miller 2023), and some studies have linked an increase in autistic traits over time to a lower IQ (Waizbard‐Bartov et al. 2021).

There seems to be a link between AS and ASD. The 15q11–q13 chromosome region affected in AS and especially the *UBE3A* gene has also been associated with ASD (Nurmi et al. 2001; Baron et al. 2006). Characteristics of AS overlap with characteristics of ASD, such as speech or communication impairments and repetitive behaviors. The majority of AS children also have sensory processing issues, such as sensory‐seeking behaviors, hyperreactivity to stimuli, and unusual interest in sensory experiences with water (Walz and Baranek 2006). Not surprisingly, a high prevalence of ASD in AS has been reported, although this prevalence was highly variable between studies, ranging from 42% to 81% (Bonati et al. 2007; Trillingsgaard and Ostengaard 2004; Peters et al. 2012; Mertz et al. 2014; Sahoo et al. 2006; Wink et al. 2015; Leader et al. 2022). This high variability can be partly explained due to differences in ASD assessment: clinician assessment only, parent report only, or combined. Previous studies on ASD in AS have shown that children with the deletion genotype exhibit more autistic traits than children with the UPD/ICD and *UBE3A* mutation genotypes (Mertz et al. 2014; Bakke et al. 2018). Furthermore, children with AS and ASD exhibit a lower cognitive developmental level, lower adaptive behavior, and lower language level compared to children with AS without ASD (Peters et al. 2004).

Little is known about the course of autistic traits in individuals with AS over time. A follow‐up study of seven individuals with the deletion genotype showed no change in autistic traits over 12 years (Mertz et al. 2014). In a one‐year follow‐up study of 42 children and young adults with AS, Peters et al. (2012) observed that, although not statistically significant, social affect scores increased over time for AS individuals with larger deletions and remained stable for AS individuals with smaller deletions. It is crucial to ascertain whether these trends persist within a larger cohort and over a longer follow‐up period. To the best of our knowledge, the longitudinal course of sensory processing difficulties in AS has not yet been studied, but cross‐sectionally, a correlation between sensory processing and age has been found (Walz and Baranek 2006).

The current study aims to investigate the effect of age on autistic traits and sensory processing issues in children with Angelman syndrome, using a large clinical cohort, a large follow‐up period, and a multi‐informant approach. In addition, we will investigate whether (changes in) autistic traits differ significantly between AS genotypes, gender, and epilepsy status. This information may inform clinicians with regard to diagnostic follow‐up procedures and may allow individuals with AS and their families to have a more precise prognosis.

## 2 Methods

### 2.1 Participants

**FIGURE 1:**
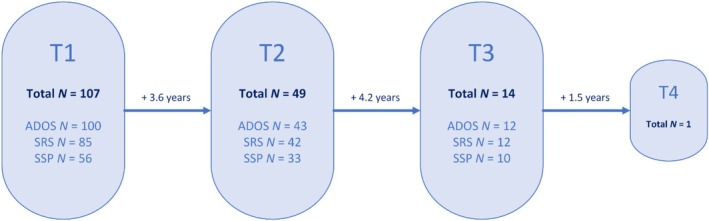
Flow chart of the repeated measurements. The time between the visits is the average of all participants. ADOS = Autism diagnostic observation schedule; SRS = social responsiveness scale; SSP = short sensory profile.

The sample comprised 107 children with molecularly confirmed AS, aged between 2.2 and 18.2 years. This sample size is relatively large considering the rarity of the syndrome. A mosaic form of AS was an exclusion criterion for this study. For descriptive purposes, Figure 1 shows the number of participants of whom we have respectively one (*n* = 58), two (*n* = 49), three (*n* = 14), or four (*n* = 1) measurements. Please note that our choice of statistical model made it possible to use all available measures in the analyses (see Section 2.4). The average time between Measurement 1 and 2 was 3.6 years, while the mean time between Measurement 2 and 3 was 4.2 years. On average, children were 7.5 years old at their first visit (*N* = 107, SD = 4.6, range 2–18), 10.8 years old at their second visit (*N* = 49, SD = 4.3, range 4–18), and 12.1 years old at their third visit (*N* = 14, SD = 3.2, range 5–18). Our choice of statistical model made it possible to analyze the effect of age on autistic traits (rather than the effect of time point on autistic traits).

### 2.2 Procedure

This prospective clinical record study used data collected in standard care. The Medical Ethics Committee at the Erasmus MC in Rotterdam, The Netherlands, has reviewed the research proposal and decided that this study did not fall within the scope of the Medical Research Involving Human Subjects Act (MEC‐2015‐203). Participants were excluded from analyses if their legal representatives stated that they did not give informed consent to use their data for scientific research. The data was collected between 2011 and 2023 at the multidisciplinary ENCORE Expertise Centre for AS at the Erasmus Medical Centre Sophia Children's Hospital in Rotterdam, the Netherlands (Bindels‐de Heus et al. 2020). All children known at the ENCORE Expertise Centre, the only AS center in the Netherlands, were invited for (neuro) psychological assessments at the department of Child‐ and Adolescent Psychiatry/Psychology. When the expertise center started in 2011, all children with AS known to us between the ages of 2 and 18 were invited for a first visit and were re‐invited every 3–4 years. Children with AS born after 2011 were invited around predetermined ages, namely around 3, 7, 11, and 15 years of age. However, not all participants were seen at these key ages. Reasons for not participating in (follow‐up) visits could be that testing was done somewhere else (closer to home) or that parents found (follow‐up) testing not necessary. If parents indicated that the timing of an appointment was not convenient, appointments could be postponed for several months or years. On the other hand, if parents indicated that they had a question or problem, appointments could also be brought forward. All appointments were utilized in the analyses, regardless of their timing (see Section 2.4).

### 2.3 Measures

#### 2.3.1 Predictors and Covariates

Age was defined as chronological age in years at the time of testing. Genotype was molecularly confirmed in accordance with the methodology described in Bindels‐de Heus et al. (2020). Genotype was classified as deletion, UPD/ICD, or pathogenic variant of the *UBE3A* gene (*UBE3A* mutation). UPD and ICD were considered together due to the small size of the ICD group and the similarity of their phenotypes (Lossie et al. 2001). Epilepsy was ascertained during a visit to the pediatric neurologist and categorized into two groups: an epilepsy group (active epilepsy or epilepsy in remission) and a no epilepsy group (never had epilepsy). Epilepsy status was maintained over time to avoid overly complex model interpretation. Gender was defined as boy or girl.

#### 2.3.2 Outcomes

The presence and severity of autistic traits were evaluated using the Autism Diagnostic Observation Schedule module 1 (ADOS; Lord et al. 2013; revised algorithms from Gotham et al. 2007), the Social Responsiveness Scale (SRS; Constantino and Gruber 2012), and the Short Sensory Profile (SSP; McIntosh et al. 1999). An elaborate description of these instruments can be found in Appendix 1. Children with AS generally have a cognitive developmental age between 12 and 27 months (Sadhwani et al. 2021; Hagenaar et al. 2024; Gentile et al. 2010). However, there is a lack of assessment tools tailored specifically for this population. Therefore, we utilized instruments suited to the developmental age of children with AS, rather than their chronological age. For all instruments, raw scores were used in the analyses, as age matched norm groups were often not available. The ADOS is a standardized observational instrument providing a total score and two subscale scores, namely social affect and restricted/repetitive behaviors (RRB). It also provides classifications of the total score into three categories: “non‐spectrum” (lowest scores), “autism spectrum disorder” and “autism” (highest scores).

The SRS is a screening questionnaire preferably completed by both a parent/caregiver and a teacher/daycare provider, independently of each other (a mean score was calculated if both informants were available). In this study, the SRS for children aged 30–48 months was completed from 2011 until 2016 (approximately half of the sample). This version consists of 65 items. From 2017 until 2023 (the other half of the sample), the SRS for children aged 18–30 months was adopted. This version is thought to be more suitable for the developmental age of AS children but consists of 50 items without available reference values. Scores of the different versions were made comparable (see Appendix 1) and version number was included as a covariate in the analyses.

The SSP is a parent‐reported questionnaire designed to assess sensory processing behaviors in everyday situations. The short version consists of 38 items, which yield a total score and seven subscale scores, namely tactile sensitivity, taste/smell sensitivity, movement sensitivity, underresponsive/seeks sensation, auditory filtering, low energy/weak, and visual/auditory sensitivity.

#### 2.3.3 Descriptive Information

This study used data collected in standard care at the department of Child and Adolescent Psychiatry/Psychology. Therefore, Diagnostic and Statistical Manual of Mental Disorders (DSM) classifications were available for descriptive purposes. DSM‐IV or ‐V (American Psychiatric Association 1994, 2013) classification was made by a child and adolescent psychiatrist and a multidisciplinary team. This diagnostic classification was based on multiple sources of information, namely hetero‐anamnestic information from the parent/caregiver (also about early social development and daily functioning), hetero‐anamnestic information from the teacher/daycare provider, a clinical psychiatric evaluation of the child, and (standardized) observational information (Autism Diagnostic Observation Schedule and observations during the Bayley assessment). This information is interpreted in light of the intellectual disability. Participants who received the diagnostic classification Pervasive Developmental Disorder Not Otherwise Specified (PDD‐NOS) according to DSM‐IV were classified within the autism spectrum.

### 2.4 Data Analyses

The statistical software R (version 4.1.2; R Foundation for Statistical Computing, Vienna, Austria) was employed to fit a mixed‐effects model (lme4; Bates et al. 2015) for the raw total score of each outcome. Fixed effects included genotype, epilepsy, and gender. A mixed‐effects model allows for the analysis of unbalanced repeated measures with missing data (Cnaan et al. 1997). Therefore, this model made it possible to use all available participants and measures in the analyses—including the participants that had just one study visit. First, the goodness of fit of a model with a linear effect of age was compared to a model with a non‐linear natural cubic splines effect of age (two or three knots). Second, several random effects were compared: random intercepts for subjects, linear random slopes for age, and non‐linear random slopes for age. Model quality was assessed using likelihood ratio tests and Bayesian information criterion (BIC); the model with the highest fit to the data was used for further analyses. A model with random intercepts and a linear effect of age best fitted the data in all analyses. Using this model, the research questions were answered by looking at the main effects of age, genotype, epilepsy, and gender. *p*‐values were adjusted for multiple comparisons using the false discovery rate (FDR; Benjamini and Hochberg 1995). Exploratory analyses included interaction effects of age and genotype (likelihood ratio tests of the model with interaction versus the model without interaction), as well as using subscale scores as dependent variables. Exploratory analyses were not adjusted for multiple comparisons. Significance level was set at *p* < 0.05 (two‐tailed). In the event of missing item scores within the SRS and SSP questionnaires, a weighted total score was calculated, with a maximum of 25% of items permitted to be missing.

### 2.5 Missing Data of Attrition

A comparison was made between children who did not participate in follow‐up visits (*n* = 58) and children from whom at least one follow‐up assessment was available (*n* = 49). Children who participated in follow‐up visits had a higher baseline SRS score (*T* = −2.48 (83), *p* = 0.015) and were more likely to have a clinician DSM classification (*χ*2 = 4.49 (1), *p* = 0.034), but showed no differences in ADOS score, SSP score, genotype, gender, epilepsy status, or socio‐economic status compared to children who did not participate in follow‐up visits (i.e., who had only one visit).

## 3 Results

### 3.1 Descriptive Characteristics

Table 1 shows the descriptive characteristics of the study sample per age group. What stands out is that the most common DSM classification in childhood was attention deficit hyperactivity disorder (ADHD; up to 37%), while the most common DSM classification in adolescence was ASD (up to 32%). Further descriptive characteristics (e.g., per study visit) are reported in Appendix 2, Table A2a–A2c. Data on race were not recorded, as this is not encouraged in the Netherlands without having a specific hypothesis on the topic. Finally, there was a significant correlation between SRS and SSP scores (*r* = −0.305; *p* = 0.039; *N* = 46), but not between ADOS and SRS scores (*r* = 0.164; *p* = 0.153; *N* = 78) or between ADOS and SSP scores (*r* = 0.112; *p* = 0.438; *N* = 50).

**TABLE 1:** Descriptive characteristics per age group.

	Frequency (percentage)			
	Age 2–5.9	Age 6–9.9	Age 10–13.9	Age 14–18.9
	(*N* = 62)	(*N* = 41)	(*N* = 34)	(*N* = 34)
Genotype				
Deletion	39 (63%)	23 (56%)	16 (47%)	20 (59%)
Non‐deletion	23 (37%)	18 (44%)	18 (53%)	14 (41%)
*Paternal uniparental disomy*	10 (16%)	10 (24%)	8 (24%)	3 (9%)
*Imprinting center defect*	2 (3%)	1 (2%)	2 (6%)	2 (6%)
*UBE3A mutation*	11 (18%)	7 (17%)	8 (24%)	9 (26%)
Epilepsy				
Yes (active or in remission)	45 (73%)	35 (85%)	32 (94%)	32 (94%)
No, never	17 (27%)	6 (15%)	2 (6%)	2 (6%)
Gender				
Girl	33 (53%)	19 (46%)	13 (38%)	16 (47%)
Boy	29 (47%)	22 (54%)	21 (62%)	18 (53%)
Socio‐economic status^a^				
Low level education^a^	0 (0%)	0 (0%)	1 (3%)	1 (3%)
Middle level education^a^	33 (53%)	21 (51%)	14 (41%)	15 (44%)
High level education^a^	20 (32%)	14 (34%)	12 (35%)	11 (32%)
Missing information	9 (15%)	6 (15%)	7 (21%)	7 (21%)
DSM classification				
None	37 (60%)	19 (46%)	16 (47%)	18 (53%)
ADHD	13 (21%)	15 (37%)	6 (18%)	4 (12%)
ASD (and previously PDD‐NOS)	8 (13%)	6 (15%)	8 (24%)	11 (32%)
Regulatory disorder (DC 0–3)	1 (2%)	1 (2%)	2 (6%)	1 (3%)
Pica	2 (3%)	0 (0%)	0 (0%)	0 (0%)
Sensory over‐responsivity disorder (ICD)	0 (0%)	0 (0%)	1 (3%)	0 (0%)
Unspecified disruptive, impulse‐control, and conduct disorder	1 (2%)	0 (0%)	1 (3%)	0 (0%)

*Note*: all repeated measures were included in this descriptive table; a minority of children may have two study visits within one age group. This was accounted for in the analyses by using mixed effects models.

Abbreviations: ADHD = attention deficit/hyperactivity disorder; ASD = Autism spectrum disorder; DC 0–3: diagnostic classification of mental health and developmental disorders of infancy and early childhood; DSM = diagnostic and statistical manual of mental disorders; ICD = International classification of diseases; PDD‐NOS = pervasive developmental disorder not otherwise specified.

^a^ Socio‐economic status was assessed using the highest educational level of the parents. Low level education was defined as no education or primary education only. Middle level education consisted of secondary education only or middle level vocational education. High level education was defined as high level vocational education, university education, or PhD education.

### 3.2 Autism Diagnostic Observation Schedule (ADOS)

**FIGURE 2:**
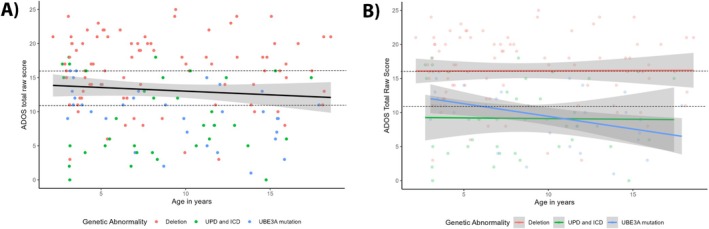
Age‐related trajectories of ADOS total raw scores. Panel (A) shows a visual representation of the main effect of age on ADOS total raw score. Panel (B) shows the interaction effect between age and genotype on the ADOS total raw score. The black line in figure A represents the average ADOS total raw score over time (not corrected for covariates), with the gray band showing the 95% confidence interval. Higher scores reflect more autistic traits. All scores above the upper dashed line are classified by the ADOS as “autism,” all scores between the two dashed lines are “ASD,” and all points below the lower dashed line are “non‐spectrum.” These classifications are based on the algorithm for children with “little to no words,” which was used for approximately 90% of participants. ADOS = Autism diagnostic observation schedule; ICD = imprinting center defect; UPD = uniparental paternal disomy.

Figure 2A shows that, on average, AS children had an ADOS total score that was classified within the ASD range. The ADOS total score did not change significantly with age. However, the deletion genotype had significantly higher ADOS scores (indicating more autistic traits) than the UPD/ICD genotypes (*B* = −6.25, *p* = 0.003) and the *UBE3A* mutation genotype (*B* = −6.09, *p* = 0.003). Epilepsy or gender did not have a significant effect. In addition, Figure 2B depicts a significant interaction effect between age and genotype, LR = 7.19 (2), *p* = 0.028. Post hoc analyses showed that scores in the deletion group stayed stable over time, whereas scores in the *UBE3A* mutation group decreased over time (*B* = −0.57, *p* = 0.011).

**FIGURE 3:**
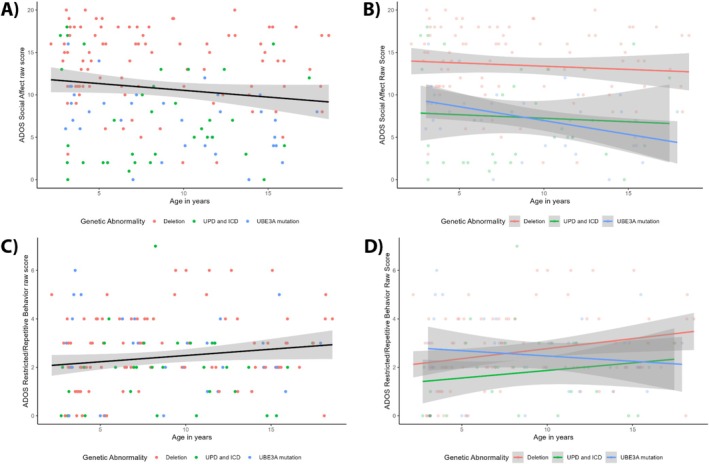
Age‐related trajectories of ADOS domain scores. Panel (A) shows the main effect of age on the ADOS social affect score, while Panel (B) displays the interaction effect between age and genotype on the ADOS social affect score. Panel (C) shows the main effect of age on the ADOS restricted/repetitive behavior score, and Panel (D) depicts the interaction between age and genotype on the ADOS restricted/repetitive behavior score. The black line in Figure A and C represents the mean ADOS scale scores over time (not corrected for covariates), with the gray band showing the 95% confidence interval. Higher scores indicate more autistic traits. ADOS = Autism diagnostic observation schedule; ICD = imprinting center defect; UPD = uniparental paternal disomy.

In Figure 3A, exploratory analyses of the ADOS subscales show that there was a significant main effect of age on the ADOS Social Affect score: social affect problems decrease as children age, *B* = −0.17, *p* = 0.047. Similar to the total score analyses, the deletion genotype had a significantly higher ADOS Social Affect score than the UPD/ICD genotypes (*B* = −5.44, *p* < 0.001) and the *UBE3A* mutation genotype (*B* = −5.93, *p* < 0.001). Gender and epilepsy did not have a significant effect. There was no significant interaction between age and genotype on the ADOS Social Affect score, LR = 4.00 (2), *p* = 0.135, although visually a similar trend as for the ADOS total scores can be seen (Figure 3B), namely that the scores for the deletion group stay stable over time while the scores for the non‐deletion groups decrease over time. From Figure 3C, it can be noted that age, gender, and epilepsy did not have a significant effect on the ADOS RRB scores. The deletion group had significantly higher RRB scores than the UPD/ICD group, *B* = −0.80, *p* = 0.030. There was no significant interaction between age and genotype on RRB scores (Figure 3D; LR = 5.58 (2), *p* = 0.062). All test statistics are reported in Appendix 3 Table A3a–A3c.

### 3.3 Social Responsiveness Scale (SRS)

**FIGURE 4:**
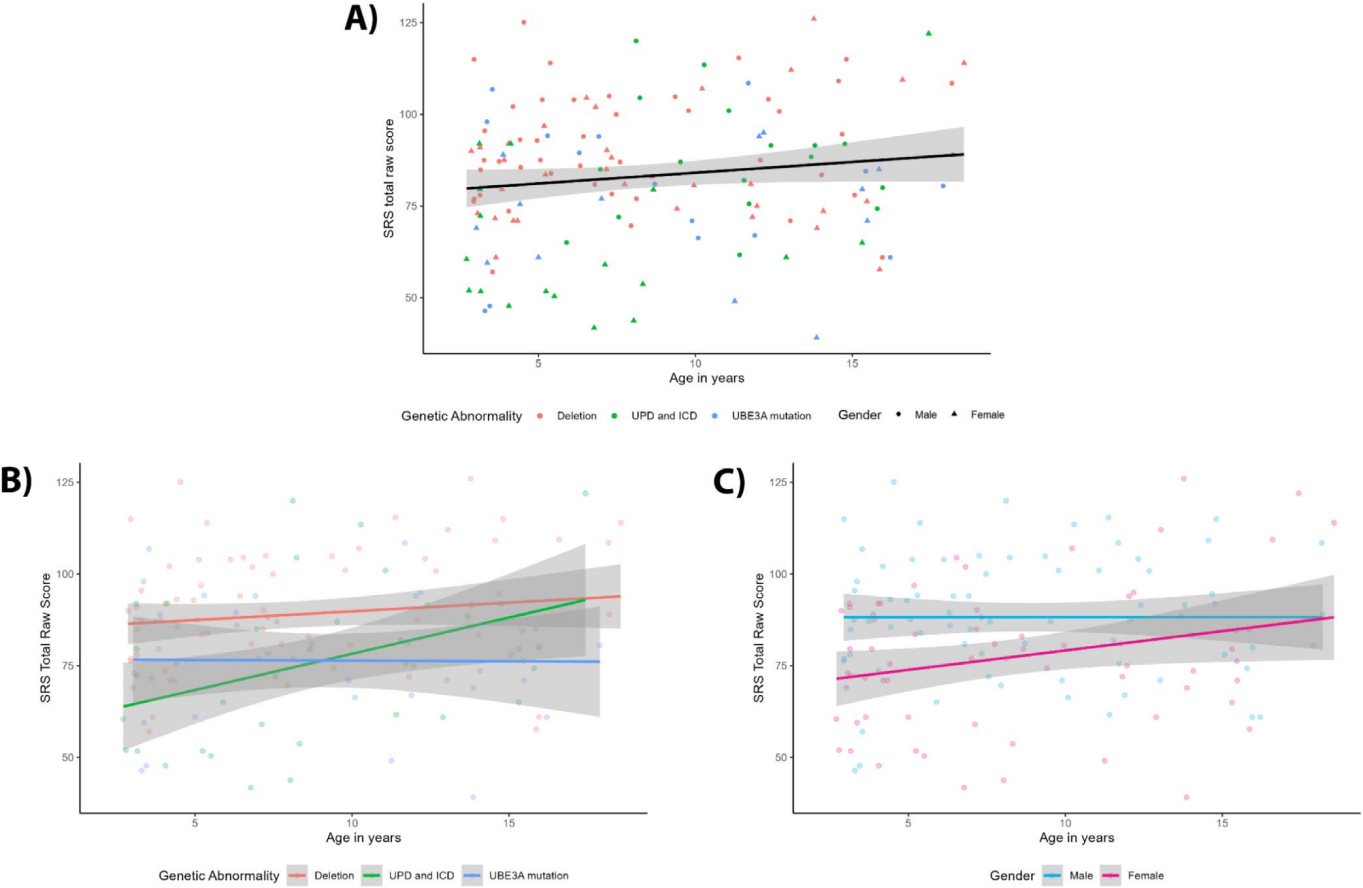
Age‐related trajectories of SRS total raw score. Panel (A) displays the main effect of age on SRS total raw score, Panel (B) shows the interaction between age and genotype on SRS total raw score, and Panel (C) depicts the interaction between age and gender on SRS total raw score. The black line in Figure (A) represents the average SRS total raw score over time (not corrected for covariates), with the gray band showing the 95% confidence interval. Higher scores indicate more autistic traits. ADOS = Autism diagnostic observation schedule; ICD = imprinting center defect; UPD = uniparental paternal disomy.

Figure 4A shows that SRS total scores did not change significantly as children age. In contrast, there was a significant effect of genotype and gender on SRS total score. Children with the deletion subtype had higher SRS total scores (indicating more autistic traits) than children with the UPD/ICD subtypes (*B* = −10.51, *p* = 0.008) and *UBE3A* mutation subtype (*B* = −11.91, *p* = 0.006). Boys had higher SRS total scores than girls, *B* = −7.81, *p* = 0.024. In addition, the SRS version number had a significant influence on the SRS total score; children scored significantly lower on the SRS 18–30 months version than on the SRS 30–48 months version (*B* = 13.99, *p* < 0.001). More test statistics can be found in Appendix 3 Table A3d. From Figure 4B,C, we conclude that there are no significant interaction effects between age and genotype (LR (2) = 2.03, *p* = 0.363), nor between age and gender (LR (1) = 1.48, *p* = 0.224).

### 3.4 Short Sensory Profile (SSP)

**FIGURE 5:**
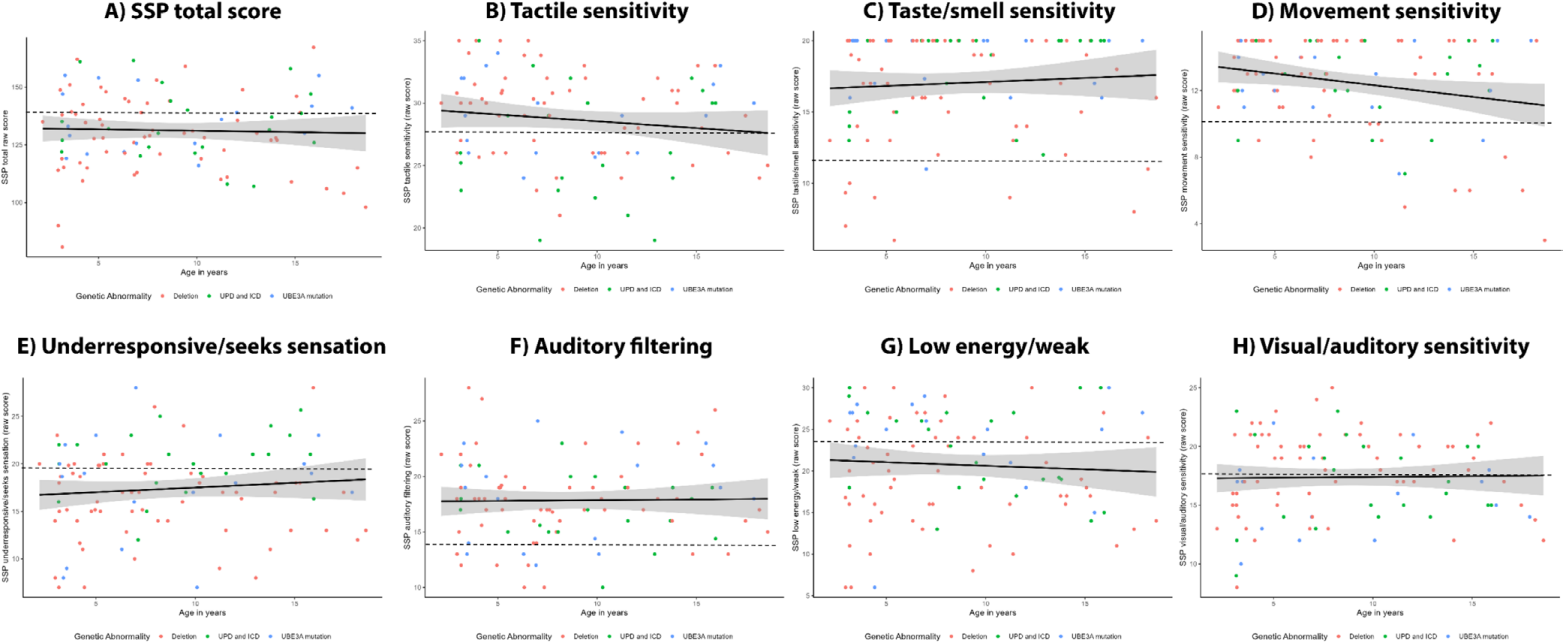
Age‐related trajectories of SSP raw total and subscales scores. The solid black line represents the mean SSP total raw score over time (not corrected for covariates), with the gray band showing the 95% confidence interval. Lower scores indicate more sensory processing problems. All scores below the dashed line are classified as “exceptionally low” in comparison to typically developing children (reference values of the SSP manual), indicating high sensory processing problems. ADOS = Autism diagnostic observation schedule; ICD = imprinting center defect; UPD = uniparental paternal disomy.

Figure 5A shows that SSP total scores were exceptionally low, indicating that AS children have significantly more sensory processing problems than children in the general population. SSP total scores did not significantly change with age. Genotype, gender, and epilepsy did not have a significant effect. Test statistics are reported in Appendix 3 Table A3e. In addition, there was no significant interaction effect between age and genotype on SSP total score, LR (2) = 0.18, *p* = 0.915.

Figure 5B–H indicate that when exploring the SSP subscales, AS children experience significantly more sensory processing problems than neurotypical children on the scales underresponsive/seeks sensation, low energy/weak, and visual/auditory sensitivity. Moreover, a significant genotype difference was found on tactile sensitivity scores: participants with the UPD/ICD genotype scored significantly lower (indicating more tactile sensitivity) than children with the *UBE3A* mutation and deletion genotypes, *B* = 3.42, *p* = 0.016 and *B* = −2.69, *p* = 0.022, respectively. Significant genotype differences were also found on underresponsive/seeks sensation scores, where participants with the deletion genotype scored significantly lower (meaning more underresponsivity/sensation seeking) than participants with the UPD/ICD and *UBE3A* mutation genotypes, *B* = 3.91, *p* = 0.004 and *B* = 3.07, *p* = 0.032, respectively. Regarding auditory filtering, there was a significant difference in gender (*B* = 2.59, *p* = 0.001), indicating that boys with AS had more problems with auditory filtering than girls with AS. There were no significant main effects of age, genotype, gender, and epilepsy on the scores of subscales taste/smell sensitivity, movement sensitivity, low energy/weak, and visual/auditory sensitivity (Appendix 3 Table A3f). Also, there was no interaction effect between age and genotype on any of the SSP subscale scores.

## 4 Discussion

In a large Dutch cohort of AS children, partially assessed longitudinally, we found a high prevalence of autistic traits and sensory processing problems as measured with the ADOS, SRS, and SSP. However, a classification of ASD with the ADOS did not automatically lead to a clinician‐derived DSM diagnosis of ASD. Furthermore, children with the deletion genotype had significantly more autistic traits on the ADOS and SRS than children with the non‐deletion genotypes, but no genotype difference was found for sensory processing problems. Boys with AS had more autistic traits than girls with AS on the SRS, although not on the ADOS. Most importantly, our findings suggest that, on average, autistic traits do not change over time in AS children. Nonetheless, there are some exceptions to this finding. The course of ADOS autistic traits over time differed significantly between genotypes: ADOS autistic traits stayed stable over time for children with the deletion genotype, whereas they decreased over time for children with the *UBE3A* genotype. In addition, when looking at the different domain scores of the ADOS, we saw that social affect problems decreased significantly as AS children aged, while restricted/repetitive behaviors stayed stable over time.

Our finding that autistic traits and sensory processing problems are highly prevalent in AS children is in line with previous research (e.g., Bonati et al. 2007; Trillingsgaard and Ostengaard 2004; Walz and Baranek 2006). However, there seems to be a discrepancy between ADOS classifications and clinician DSM classifications: on the first study visit, 76% of participants had an ADOS score that fell within the ASD range, but only 16% of children received a clinician DSM classification of ASD. In many cases, the autistic traits were regarded as being in proportion to the ID. This finding contributes to the ongoing debate surrounding the interpretation of autistic traits in AS. Some researchers have proposed that autistic traits in AS may be solely attributable to their level of ID (Moss and Howlin 2009; Trillingsgaard and Ostengaard 2004). Diagnostic instruments such as the ADOS have been found to overdiagnose ASD in individuals with severe ID (Sappok et al. 2013). For children with a developmental age under or around 12 months, many social and communicative skills that are used in the ADOS to measure ASD have not yet developed due to their developmental delay, which is consistent with the development observed in other domains. In addition, several studies have indicated social interest, social approach behaviors, and social enjoyment as relative strengths for the majority of AS children (Trillingsgaard and Ostengaard 2004; Oliver et al. 2007; Moss et al. 2013; Lubbers et al. 2022).

Furthermore, our findings demonstrate that children with the deletion genotype exhibit significantly higher levels of autistic traits on the ADOS and SRS than children with the non‐deletion genotypes, while no genotype difference was found for sensory processing problems. This pattern is in line with previous research (Mertz et al. 2014; Bakke et al. 2018; Walz and Baranek 2006). It is unsurprising that different patterns were identified for autistic traits and sensory processing, as these represent distinct constructs, with autistic traits encompassing a broader range of characteristics beyond sensory processing alone. Unexpectedly, we found that children with the UPD/ICD genotype had significantly more tactile sensitivity than children with the deletion and *UBE3A* genotypes. This may be because children with the UPD/ICD genotypes generally have a higher cognitive developmental level (Keute et al. 2020), enabling them to learn by paired association. In combination with tactile sensitivity, this could lead to anticipation anxiety and avoidance, making tactile sensitivity problems more visible.

We found that boys with AS had more autistic traits than girls with AS on the SRS, but not on the ADOS. When exploring domains of sensory processing, we found that boys with AS had more problems with auditory filtering than girls with AS. In individuals with intellectual disabilities, the male‐to‐female ratio of ASD is estimated to be around 2:1 (Saure et al. 2023). Possibly, our SRS results reflect a genuine gender difference in autistic traits in AS, similar to those in individuals with ID. Alternatively, it could reflect a gender bias in the perception of parents and teachers, in the sense that behavior is more often recognized as autistic in boys than in girls (Lai et al. 2015). The discrepancy in findings between the SRS and ADOS relates to the low correlation between these measures reported in previous research (Reszka et al. 2014), and may stem from differences in measurement type (parent/teacher reported versus clinician observed). For example, the SRS may be more reflective of everyday functioning, while the ADOS may be more objective.

Our results demonstrate that autistic traits as measured by the ADOS and SRS remain stable throughout childhood and adolescence. This finding confirms previous research in AS using a smaller sample size and shorter follow‐up time (Peters et al. 2012; Mertz et al. 2014). However, we uniquely extend these findings by showing that the course of ADOS autistic traits over time differed significantly between genotypes: ADOS autistic traits stayed stable over time for children with the deletion genotype, whereas ADOS autistic traits decreased over time for children with the *UBE3A* genotype. This pattern may be attributed to differing developmental levels, as previous research showed that a decrease in autistic traits over time in the general ASD population is associated with higher IQ (Waizbard‐Bartov and Miller 2023), and children with non‐deletions typically exhibit a higher developmental level than children with deletions (Keute et al. 2020). When exploring the ADOS domain scores, we observed that social affect problems decrease significantly as children age, but restricted and repetitive behaviors remain stable over time. Previous studies have demonstrated that restricted/repetitive behaviors are observed in all individuals with AS, regardless of whether they have been diagnosed with ASD. This suggests that these behaviors are associated with (severe) ID, rather than being specific to ASD (Bonati et al. 2007). Finally, our results indicate that sensory problems do not change significantly with age, consistent with most studies in the general population (e.g., Perez Repetto et al. 2017). An earlier cross‐sectional study (Walz and Baranek 2006) identified a correlation between sensory processing and age in AS. The current study does not confirm this result.

The current study has several strengths. First, it employs a large clinical sample, with up to three repeated measurements taken over an extended follow‐up period of approximately 4 years between subsequent measurements. Second, it utilizes three different measurement instruments to assess autistic traits, including both clinician assessments (ADOS) and parent/teacher reports (SRS and SSP). Third, it allows for comparison with clinician DSM diagnoses, based on the state‐of‐the‐art multi‐informant, multidisciplinary model. The current study is limited by the use of measurement instruments that were not designed for this specific population or for children with a severe developmental delay, as these instruments are not yet available. This could have impacted the reliability and validity of the measurements, possibly leading to an overestimation of autistic traits. Further, this study utilized different versions of the SRS, which significantly influenced the results: higher scores were found for the SRS 30–48 months version (used prior to 2016) and lower scores for the SRS 18–30 months version (used after 2016). This (partly) longitudinal study employed data from clinical care from 2011 until 2023; thus, changes in questionnaire version are inherent to the study design. The SRS 18–30 months version was more suitable for the developmental age of AS children and therefore probably gives a more realistic view of autistic traits in this population. We attempted to minimize bias by incorporating version number as a covariate in our analyses. A third limitation is that this study is only partially longitudinal, as there were 58 children who did not participate in follow‐up visits. Future research with more follow‐up visits may focus on within‐subject variation. In the current study, children who participated in follow‐up visits had a higher baseline SRS score and were more likely to have a clinician DSM classification than children who had only one study visit. While all children were invited for follow‐up clinical appointments, perhaps experiencing behavioral problems was a reason for accepting the invitation. This may be a potential bias, since it may have prevented us from measuring a decline in autistic traits. Fourth, the ADOS was conducted by several different (trained) psychologists, which could have led to inter‐observer variability. Fifth, clinician DSM classification may be influenced by local policies regarding educational and other support opportunities for the child, which may differ per country. Also, the consideration of the impact of the symptoms on life functioning while making a diagnosis may differ per country, and future studies could include an objective measure of daily functioning. Finally, the SSP “low energy/weak” scale score is possibly influenced by motor problems in AS and therefore does not solely reflect sensory processing problems.

This study poses significant implications for clinical practice. Our results can be used to provide more precise information on autism traits for parents of AS children. Furthermore, it is important to be cautious when interpreting ADOS results in this population, as this instrument may overestimate ASD traits in AS due to their severe ID. Future research on autistic traits in AS could test the suitability of the adapted version of the ADOS (Bal et al. 2020) for individuals with AS. Our results indicate that although autistic traits remain stable over time when the whole group is considered, there is a change in autistic traits over time for specific genetic subgroups (*UBE3A* mutation genotype) and for specific domains (social affect). The ADOS can be used to identify strengths and weaknesses in the social contact of the child, which could inform personalized strategies for social learning and adaptations of the environment to the child's needs. Our results show that sensory processing problems are exceptionally high in AS children and do not decrease as children age. Consequently, incorporating the evaluation of sensory processing in clinical practice is of paramount importance, particularly given that a significant proportion of AS children are unable to report on their own sensory preferences. Individual sensory processing profiles can inform personalized behavioral interventions, as behavioral problems can be an expression of sensory overload or be a way to seek sensation. Minimizing or maximizing certain sensory stimuli in the daily environment of these individuals can therefore influence their behavior, emotions, and ultimately their quality of life.

## 5 Conclusion

We show a high prevalence of autistic traits and sensory processing issues in AS, where children with the deletion genotype exhibit more autistic traits. Overall, autistic traits remained stable over time, except that ADOS scores significantly decreased in children with the *UBE3A* mutation genotype and in the social affect domain for the entire group. Therefore, incorporating the assessment of autistic traits and sensory processing into clinical practice for AS is important to inform adaptations of the environment to meet the child’s needs. In addition, clinicians and researchers should be aware of the risk for overestimating ASD traits in AS on the basis of the ADOS. Diagnosis of ASD in AS should always be made based on a combination of diagnostic instruments, heteroanamnestic information from different sources, and expert clinical opinion in a multidisciplinary team.

## Conflicts of Interest

Marie‐Claire de Wit is the Erasmus MC study site leader for the Roche Tangelo study, for which the hospital received funding. The hospital also received compensation from Roche and Jazz Pharmaceuticals for giving advice. All other authors have no conflicts of interest to declare.

## Supporting information


**Data S1.** Supporting Information.

## Data Availability

The data that support the findings of this study are available on request from the corresponding author. The data are not publicly available due to privacy or ethical restrictions.
