# Supplementary material for: Age‐Related Trajectories of Autistic Traits in Children With Angelman Syndrome
Source: Autism Res. 2025 Mar 21;18(4):870–80. doi: 10.1002/aur.70017 (PMC12015797; doi:10.1002/aur.70017)
Supplement: Supplementary file 1 — Data S1. Supporting Information. [file AUR-18-870-s001.docx]

**Appendix 1**

**Measures**

*Outcomes*

The ADOS is a standardized instrument with which a certified clinician evokes specific social situations and observes and scores how the child reacts. Different modules can be chosen depending on language skills of the child. In the current study, Module 1 was employed, as all participants exhibited limited or no verbal speech. The ADOS provides a total score and two subscale scores, namely social affect and restricted/repetitive behaviors (RRB). Some participants were assessed using an older version of the ADOS (the ADOS-G), and the scores were recoded to align with the new version. The ADOS also provides classifications of the raw total score into three categories: ‘non-spectrum’ (lowest scores), ‘autism spectrum disorder’ and ‘autism’ (highest scores). Age-matched norm groups were not available for children aged 15 years and older, therefore raw scores were used in the analyses. Norm scores and classifications were however added to the descriptive statistics to facilitate interpretation.

The SRS is a screening questionnaire for autistic traits that is preferably completed by both a parent/caregiver and a teacher/daycare provider, independently of each other. Combining the parent- and teacher-reported SRS improves prediction of an ASD classification (Duvekot et al., 2015). The SRS was filled out by both the parent and the teacher in 54% of participants, in which case the mean of the two informants total scores was calculated. In the remaining 46% of cases, the available score was used. In these instances, the score of the parent/caregiver was used in 73% of cases, while the score of the teacher/daycare provider was used in 27% of cases. It should be noted that different versions of the SRS exist for different age groups. In this study, the SRS for children aged 30-48 months was completed from 2011 until 2016 (approximately half of the sample). This version consists of 65 items, resulting in a total score and five subscale scores, for which reference values are available. From 2017 until 2023 (the other half of the sample), the SRS for children aged 18-30 months was adopted. This version is thought to be more suitable for the developmental age of children with AS, and is therefore likely to provide a more realistic view of autistic traits in this group. However, this version consists of 50 items, and no reference values are available. In order to make the scores of the different versions comparable, the total score of the 18-30 months version was multiplied by 1,3265 (to account for the number of items). The raw total scores were employed in the analyses. To account for any remaining bias, the version number (18-30 months versus 30-48 months) was included as a covariate in the analyses.

The SSP is a parent-reported questionnaire designed to assess sensory processing in everyday situations. The short version consists of 38 items, which yield a total score and seven subscale scores, namely tactile sensitivity, taste/smell sensitivity, movement sensitivity, underresponsive/seeks sensation, auditory filtering, low energy/weak, and visual/auditory sensitivity. Raw scores were employed in the analyses. Parents of children who reported sensory processing as a problem on forehand were occasionally given the longer version of the Sensory Profile (Dunn, 1994) to facilitate more comprehensive individual diagnostics. As all items of the Short Sensory Profile overlap with the longer Sensory Profile, the data from the longer Sensory Profile was recoded to align with the short version.

Dunn, W. (1994). Performance of typical children on the Sensory Profile: An item analysis. *American Journal of*

*Occupational Therapy, 48*, 967–974

Duvekot, J., van der Ende, J., Verhulst, F. C., & Greaves-Lord, K. (2015). The Screening Accuracy of the Parent and

Teacher-Reported Social Responsiveness Scale (SRS): Comparison with the 3Di and ADOS. *Journal of Autism and Developmental Disorders, 45*, 1658–1672. https://doi.org/10.1007/s10803-014-2323-3

**Appendix 2**

Table A2a

*Descriptive characteristics per age group (continuous variables)*

|  | **Age 2 to 5.9** | | | **Age 6 to 9.9** | | |
| --- | --- | --- | --- | --- | --- | --- |
|  | **(ADOS *N* = 58, SRS *N* = 52, SSP *N* = 36)** | | | **(ADOS *N* = 39, SRS *N* = 36, SSP *N* = 29)** | | |
|  | **Raw score** | **Norm score** | **Qualitative** | **Raw score** | **Norm score** | **Qualitative** |
|  | **Mean (*SD*)** | **Mean (*SD*)** | **Description** | **Mean (*SD*)** | **Mean (*SD*)** | **Description** |
| **Age** | 3.88 (0.88) | - | - | 7.77 (1.12) | - | - |
| **ADOS** |  |  |  |  |  |  |
| - Total score | 14.07 (5.66) | CSS = 5.02 (2.02) | 21% non-spectrum | 12.97 (6.57) | CSS = 4.59 (2.14) | 31% non-spectrum |
| *- Social affect* | 12.10 (5.32) |  | 33% ASD | 10.15 (5.83) |  | 33% ASD |
| *- Restricted/repetitive behavior* | 1.97 (1.69) |  | 46% autism | 2.82 (1.45) |  | 36% autism |
| **SRS** |  |  |  |  |  |  |
| - Total score | 79.82 (18.74) | - | - | 84.31 (17.18) | - | - |
| **SSP** |  |  |  |  |  |  |
| - Total score | 131.41 (17.90) | Z = -2.45 (1.15) | Except. Low | 134.10 (13.56) | Z = -2.28 (0.87) | Except. Low |
| *- Tactile sensitivity* | 29.88 (3.36) | Z = -1.23 (1.24) | Low average | 28.24 (4.33) | Z = -1.83 (1.60) | Below Average |
| *- Taste/smell sensitivity* | 16.17 (4.16) | Z = -0.56 (1.34) | Average | 18.23 (2.58) | Z = -0.11 (0.83) | Average |
| *- Movement sensitivity* | 13.06 (1.78) | Z = -0.44 (0.94) | Average | 13.02 (2.16) | Z = -0.46 (1.14) | Average |
| *- Underresponsive/seeks sensation* | 16.80 (4.67) | Z = -2.56 (0.93) | Except. Low | 17.93 (4.44) | Z = -2.33 (0.89) | Except. Low |
| *- Auditory filtering* | 18.59 (3.70) | Z = -0.98 (0.79) | Low average | 16.49 (3.67) | Z = -1.43 (0.78) | Below Average |
| *- Low energy/weak* | 20.71 (6.98) | Z = -3.00 (2.58) | Except. Low | 21.75 (5.51) | Z = -2.61 (2.04) | Except. Low |
| *- Visual/auditory sensitivity* | 16.92 (4.07) | Z = -2.25 (1.51) | Except. Low | 18.35 (3.53) | Z = -1.72 (1.31) | Below Average |
|  | **Age 10 to 13.9** | | | **Age 14 to 18.9** | | |
|  | **(ADOS *N* = 29, SRS *N* = 28, SSP *N* = 17)** | | | **(ADOS *N* = 30, SRS *N* = 24, SSP *N* = 18)** | | |
|  | **Raw score** | **Norm score** | **Qualitative** | **Raw score** | **Norm score** | **Qualitative** |
|  | **Mean (*SD*)** | **Mean (*SD*)** | **Description** | **Mean (*SD*)** | **Mean (*SD*)** | **Description** |
| **Age** | 11.91 (1.13) | - | - | 15.99 (1.33) | - | - |
| **ADOS** |  |  |  |  |  |  |
| - Total score | 11.69 (6.24) | CSS = 4.28 (2.19) | 48% non-spectrum | 13.10 (5.73) | CSS = 4.63 (1.92) | 33% non-spectrum |
| *- Social affect* | 9.07 (5.17) |  | 21% ASD | 10.53 (5.23) |  | 23% ASD |
| *- Restricted/repetitive behavior* | 2.62 (1.66) |  | 31% autism | 2.57 (1.41) |  | 43% autism |
| **SRS** |  |  |  |  |  |  |
| - Total score | 85.94 (21.38) | - | - | 86.02 (18.54) | - | - |
| **SSP** |  |  |  |  |  |  |
| - Total score | 125.33 (12.19) | Z = -2.84 (0.78) | Exept. Low | 132.03 (19.77) | Z = -2.41 (1.27) | Exept. Low |
| *- Tactile sensitivity* | 26.31 (3.34) | Z = -2.55 (1.24) | Exept. Low | 29.47 (2.85) | Z = -1.38 (1.06) | Below Average |
| *- Taste/smell sensitivity* | 16.65 (3.53) | Z = 0.40 (1.14) | Average | 17.18 (3.75) | Z = 0.23 (1.21) | Average |
| *- Movement sensitivity* | 11.65 (3.24) | Z = -1.19 (1.70) | Low Average | 11.47 (4.03) | Z = -1.28 (2.12) | Low Average |
| *- Underresponsive/seeks sensation* | 16.47 (5.14) | Z = -2.63 (1.03) | Exept. Low | 18.44 (4.45) | Z = -2.23 (0.89) | Exept. Low |
| *- Auditory filtering* | 17.59 (3.55) | Z = -1.19 (0.76) | Low Average | 18.79 (3.90) | Z = -0.94 (0.83) | Average |
| *- Low energy/weak* | 19.84 (5.35) | Z = -3.32 (1.98) | Exept. Low | 20.11 (6.55) | Z = -3.22 (2.43) | Exept. Low |
| *- Visual/auditory sensitivity* | 17.28 (2.52) | Z = -2.12 (0.93) | Exept. Low | 16.88 (3.06) | Z = -2.27 (1.13) | Exept. Low |

Note: all repeated measures were included in this descriptive table, a minority of children may have two study visits within one age group (i.e., if they had one measure at 6 years old and another measure at 8 years old). This was accounted for in the longitudinal analyses by using mixed effects models for repeated measures. For the ADOS, higher raw and CSS scores reflect more autistic traits. On the SRS, higher scores indicate more autistic traits. The opposite is true for the SSP, where lower raw scores reflect more sensory processing problems.

Abbreviations: ADOS = Autism Diagnostic Observation Schedule; ASD = Autism Spectrum Disorder; Behav. = behavior; CSS = Calibrated Severity Score; Except. = exceptionally; SD = Standard Deviation; SRS = Social Responsiveness Scale; SSP = Short Sensory Profile; Underresp. = underresponsive.

Table A2b

*Descriptive characteristics per study visit (categorical variables)*

|  | **Frequency (percentage)** | | |
| --- | --- | --- | --- |
|  | **T1 (*N*=107)** | **T2 (*N*=49)** | **T3**  **(*N*=14)** |
| **Genotype** |  |  |  |
| -Deletion | 66 (62%) | 26 (53%) | 6 (43%) |
| -Non-deletion | 41 (38%) | 23 (47%) | 8 (57%) |
| *-Paternal uniparental disomy (UPD)* | *17 (16%)* | *10 (20%)* | *3 (21%)* |
| *-Imprinting center defect (IC)* | *3 (3%)* | *3 (6%)* | *1 (7%)* |
| *-UBE3A mutation* | *21 (20%)* | *10 (20%)* | *4 (29%)* |
| **Epilepsy** |  |  |  |
| -Yes (active or in remission) | 89 (83%) | 44 (90%) | 11 (79%) |
| -No, never | 18 (17%) | 5 (10%) | 3 (21%) |
| **Gender** |  |  |  |
| -Girl | 50 (47%) | 25 (51%) | 6 (43%) |
| -Boy | 57 (53%) | 24 (49%) | 8 (57%) |
| **Socio-economic status*** |  |  |  |
| -Low level education* | 1 (1%) | 1 (2%) | 0 (0%) |
| -Middle level education* | 51 (48%) | 24 (49%) | 8 (57%) |
| -High level education* | 33 (31%) | 17 (35%) | 6 (43%) |
| -Missing information | 22 (21%) | 7 (14%) | 0 (0%) |
| **DSM Classification** |  |  |  |
| -None | 62 (58%) | 23 (47%) | 4 (29%) |
| -ADHD | 21 (20%) | 12 (25%) | 5 (36%) |
| -ASD (including previously PDD-NOS) | 17 (16%) | 12 (25%) | 4 (29%) |
| -Regulatory Disorder (DC 0-3) | 3 (3%) | 1 (2%) | 1 (7%) |
| -Pica | 2 (2%) | 0 (0%) | 0 (0%) |
| -Sensory over-responsivity disorder (ICD) | 1 (1%) | 0 (0%) | 0 (0%) |
| -Unspecified disruptive, | 1 (1%) | 1 (2%) | 0 (0%) |
| impulse-control, and conduct disorder |  |  |  |

*Socio-economic status was assessed using the highest educational level of the parents. Low level education was defined as no education or primary education only. Middle level education consisted of secondary education only or middle level vocational education. High level education was defined as high level vocational education, university education, or PhD education.

Abbreviations: ADHD = Attention Deficit/Hyperactivity Disorder; ASD = Autism Spectrum Disorder; DC 0-3: Diagnostic Classification of Mental Health and Developmental Disorders of Infancy and Early Childhood; DSM = Diagnostic and Statistical Manual of Mental Disorders; ICD = International Classification of Diseases; PDD-NOS = Pervasive Developmental Disorder Not Otherwise Specified.

Table A2c

*Descriptive characteristics per study visit (continuous variables)*

|  | **T1 (ADOS *N*=100, SRS *N*=85, SSP *N*=58)** | | | **T2 (ADOS *N*=43, SRS *N*=42, SSP *N*=33)** | | | **T3 (ADOS *N*=12, SRS *N*=13, SSP *N*=10)** | | |
| --- | --- | --- | --- | --- | --- | --- | --- | --- | --- |
|  | **Raw score** | **Norm score** | **Qualitative** | **Raw score** | **Norm score** | **Qualitative** | **Raw score** | **Norm score** | **Qualitative** |
|  | **Mean (*SD*)** | **Mean (*SD*)** | **Description** | **Mean (*SD*)** | **Mean (*SD*)** | **Description** | **Mean (*SD*)** | **Mean (*SD*)** | **Description** |
| **Age** | 7.51 (4.62) | - | - | 10.78 (4.33) | - | - | 12.11 (3.25) | - | - |
| **ADOS** |  |  |  |  |  |  |  |  |  |
| - Total score | 13.70 (5.57) | CSS = 4.84 (1.91) | 24% non-spectrum | 12.93 (6.55) | CSS = 4.65 (2.28) | 35% non-spectrum | 10.50 (6.79) | CSS = 4.00 (2.30) | 67% non-spectrum |
| *- Social affect* | 11.44 (5.07) |  | 32% ASD | 10.21 (5.85) |  | 28% ASD | 7.75 (6.03) |  | 8% ASD |
| *- Restricted/repetitive behav.* | 2.26 (1.62) |  | 44% autism | 2.72 (1.56) |  | 37% autism | 2.75 (1.42) |  | 25% autism |
| **SRS** |  |  |  |  |  |  |  |  |  |
| - Total score | 85.30 (18.01) | - | - | 81.72 (17.56) | - | - | 77.66 (25.41) | - | - |
| **SSP** |  |  |  |  |  |  |  |  |  |
| - Total score | 129.10 (15.96) | Z = -2.60 (1.02) | Except. low | 135.31 (16.46) | Z = -2.20 (1.06) | Except. low | 127.07 (13.54) | Z = -2.73 (0.87) | Except. low |
| *- Tactile sensitivity* | 28.38 (3.69) | Z = -1.78 (1.37) | Below average | 29.43 (3.99) | Z = -1.40 (1.48) | Below average | 27.30 (3.07) | Z = -2.19 (1.14) | Except. low |
| *- Taste/smell sensitivity* | 16.51 (3.90) | Z = -0.45 (1.26) | Average | 17.53 (3.31) | Z = -0.12 (1.07) | Average | 18.00 (2.79) | Z = 0.03 (0.90) | Average |
| *- Movement sensitivity* | 12.72 (2.38) | Z = -0.62 (1.25) | Average | 12.44 (2.91) | Z = -0.77 (1.53) | Average | 11.40 (3.86) | Z = -1.32 (2.03) | Below average |
| *- Underresp./seeks sensation* | 16.67 (4.59) | Z = -2.59 (0.92) | Except. low | 18.93 (4.22) | Z = -2.13 (0.84) | Except. low | 15.53 (5.02) | Z = -2.81 (1.00) | Except. low |
| *- Auditory filtering* | 17.84 (3.70) | Z = -1.13 (0.79) | Low average | 17.94 (4.01) | Z = -1.12 (0.85) | Low average | 17.49 (3.63) | Z = -1.22 (0.77) | Low average |
| *- Low energy/weak* | 19.89 (6.58) | Z = -3.30 (2.44) | Except. low | 21.96 (5.51) | Z = -2.53 (2.04) | Except. low | 20.90 (6.08) | Z = -2.93 (2.25) | Except. low |
| *- Visual/auditory sensitivity* | 17.48 (3.70) | Z = -2.05 (1.37) | Except. low | 17.50 (3.31) | Z = -2.04 (1.22) | Except. low | 16.20 (3.33) | Z = -2.52 (1.23) | Except. low |

For the ADOS, higher raw and CSS scores reflect more autistic traits. On the SRS, higher scores indicate more autistic traits. The opposite is true for the SSP, where lower raw scores reflect more sensory processing problems.

Abbreviations: ADOS = Autism Diagnostic Observation Schedule; ASD = Autism Spectrum Disorder; Behav. = behavior; CSS = Calibrated Severity Score; Except. = exceptionally; SD = Standard Deviation; SRS = Social Responsiveness Scale; SSP = Short Sensory Profile; Underresp. = underresponsive.

**Appendix 3**

Table A3a

*Main effects on ADOS total raw score*

|  | **B** | ***p*-value** | **Adjusted *p*** |
| --- | --- | --- | --- |
| **Age** | -0.13 | .179 | .269 |
| **Genotype** |  |  |  |
| - Deletion vs UPD/ICD | -6.25 | <.001* | .003* |
| - Deletion vs UBE3A mutation | -6.09 | <.001* | .003* |
| - UPD/ICD vs UBE3A mutation | 0.16 | .912 | .912 |
| **Gender** | -0.75 | .429 | .429 |
| **Epilepsy** | -0.26 | .851 | .851 |

Abbreviations: ICD = Imprinting Center Defect; UPD = Uniparental Paternal Disomy.

Table A3b

*Interaction age * genotype on ADOS total raw score*

|  | **B** |  | ***p*-value** |
| --- | --- | --- | --- |
| **Age * Genotype interaction** |  |  |  |
| - Deletion versus UPD/ICD | -0.37 |  | .135 |
| - Deletion versus UBE3A mutation | -0.57 |  | .011* |
| - UPD/ICD versus UBE3A mutation | -0.19 |  | .491 |

Abbreviations: ICD = Imprinting Center Defect; UPD = Uniparental Paternal Disomy.

Table A3c

*Main effects on ADOS domain scores*

| **ADOS Social Affect Score** | | |
| --- | --- | --- |
|  |  |  |
|  | **B** | ***p*-value** |
| **Age** | -0.17 | .047* |
| **Genotype** |  |  |
| - Deletion versus UPD/ICD | -5.44 | <.001* |
| - Deletion versus UBE3A mutation | -5.93 | <.001* |
| - UPD/ICD versus UBE3A mutation | -0.48 | .709 |
| **Gender** | -0.42 | .624 |
| **Epilepsy** | -0.05 | .970 |
| **ADOS Restricted/Repetitive Behavior Score** | | |
|  |  |  |
|  | **B** | ***p*-value** |
| **Age** | 0.04 | .133 |
| **Genotype** |  |  |
| - Deletion versus UPD/ICD | -0.80 | .030* |
| - Deletion versus UBE3A mutation | -0.14 | .702 |
| - UPD/ICD versus UBE3A mutation | 0.66 | .119 |
| **Gender** | -0.35 | .206 |
| **Epilepsy** | 0.27 | .505 |

Abbreviations: ICD = Imprinting Center Defect; UPD = Uniparental Paternal Disomy.

Table A3d

*Main effects on SRS total raw score*

|  | **B** | ***p*-value** | **Adjusted *p*** |
| --- | --- | --- | --- |
| **Age** | 0.54 | .086 | .258 |
| **Genotype** |  |  |  |
| - Deletion versus UPD/ICD | -10.51 | .005* | .008* |
| - Deletion versus UBE3A mutation | -11.91 | .004* | .006* |
| - UPD/ICD versus UBE3A mutation | -1.40 | .749 | .912 |
| **Gender** | -7.81 | .008* | .024* |
| **Epilepsy** | 3.01 | .473 | .851 |
| **SRS version** | 13.99 | <.001* |  |

Abbreviations: ICD = Imprinting Center Defect; UPD = Uniparental Paternal Disomy.

Table A3e

*Main effects on SSP total raw score*

|  | **B** | ***p*-value** | **Adjusted *p*** |
| --- | --- | --- | --- |
| **Age** | 0.19 | .564 | .564 |
| **Genotype** |  |  |  |
| - Deletion versus UPD/ICD | 3.58 | .493 | .493 |
| - Deletion versus UBE3A mutation | 9.87 | .074 | .074 |
| - UPD/ICD versus UBE3A mutation | 6.30 | .318 | .912 |
| **Gender** | 5.73 | .141 | .212 |
| **Epilepsy** | 2.99 | .594 | .851 |

Abbreviations: ICD = Imprinting Center Defect; UPD = Uniparental Paternal Disomy.

Table A3f

*Main effects on SSP scale scores*

| **SSP Tactile Sensitivity** | | |  |  |  |
| --- | --- | --- | --- | --- | --- |
|  | | |  | | |
|  | **B** | ***p-value*** | |  |  |
| **Age** | -0.05 | .491 | |  |  |
| **Genotype** |  |  | |  |  |
| - Deletion versus UPD/ICD | -2.69 | .022* | |  |  |
| - Deletion versus UBE3A mutation | 0.73 | .548 | |  |  |
| - UPD/ICD versus UBE3A mutation | 3.42 | .016* | |  |  |
| **Gender** | 1.04 | .229 | |  |  |
| **Epilepsy** | 0.83 | .506 | |  |  |
| **SSP Taste/Smell Sensitivity** | | |  | | |
|  |  |  |  | | |
|  | **B** | ***p-value*** | |  |  |
| **Age** | 0.12 | .082 | |  |  |
| **Genotype** |  |  | |  |  |
| - Deletion versus UPD/ICD | 1.11 | .361 | |  |  |
| - Deletion versus UBE3A mutation | 1.67 | .188 | |  |  |
| - UPD/ICD versus UBE3A mutation | 0.55 | .704 | |  |  |
| **Gender** | 0.30 | .745 | |  |  |
| **Epilepsy** | -0.37 | .773 | |  |  |
| **SSP Movement Sensitivity** | | |  | | |
|  |  |  |  | | |
|  | **B** | ***p-value*** | |  |  |
| **Age** | -0.09 | .105 | |  |  |
| **Genotype** |  |  | |  |  |
| - Deletion versus UPD/ICD | -0.39 | .640 | |  |  |
| - Deletion versus UBE3A mutation | -0.10 | .908 | |  |  |
| - UPD/ICD versus UBE3A mutation | 0.29 | .774 | |  |  |
| **Gender** | -0.41 | .517 | |  |  |
| **Epilepsy** | -0.61 | .498 | |  |  |
| **SSP Underreactive/Seeks Sensation** | | |  | | |
|  |  |  |  | | |
|  | **B** | ***p-value*** | |  |  |
| **Age** | 0.09 | .361 | |  |  |
| **Genotype** |  |  | |  |  |
| - Deletion versus UPD/ICD | 3.91 | .004* | |  |  |
| - Deletion versus UBE3A mutation | 3.07 | .032* | |  |  |
| - UPD/ICD versus UBE3A mutation | -0.84 | .603 | |  |  |
| **Gender** | 1.39 | .162 | |  |  |
| **Epilepsy** | 2.00 | .170 | |  |  |
| **SSP Auditory Filtering** | | |  | | |
|  |  |  |  | | |
|  | **B** | ***p-value*** | |  |  |
| **Age** | 0.01 | .898 | |  |  |
| **Genotype** |  |  | |  |  |
| - Deletion versus UPD/ICD | -0.99 | .923 | |  |  |
| - Deletion versus UBE3A mutation | 1.63 | .151 | |  |  |
| - UPD/ICD versus UBE3A mutation | 1.73 | .170 | |  |  |
| **Gender** | 2.59 | .001* | |  |  |
| **Epilepsy** | 2.16 | .065 | |  |  |
| **SSP Low Energy/Weak** | | |  | | |
|  |  |  |  | | |
|  | **B** | ***p-value*** | |  |  |
| **Age** | 0.01 | .955 | |  |  |
| **Genotype** |  |  | |  |  |
| - Deletion versus UPD/ICD | 2.56 | .179 | |  |  |
| - Deletion versus UBE3A mutation | 3.61 | .080 | |  |  |
| - UPD/ICD versus UBE3A mutation | 1.05 | .649 | |  |  |
| **Gender** | .024 | .867 | |  |  |
| **Epilepsy** | -1.10 | .596 | |  |  |
| **SSP Visual/Auditory Sensitivity** | | |  | | |
|  |  |  |  | | |
|  | **B** | ***p-value*** | |  |  |
| **Age** | -0.01 | 0.885 | |  |  |
| **Genotype** |  |  | |  |  |
| - Deletion versus UPD/ICD | -0.37 | 0.714 | |  |  |
| - Deletion versus UBE3A mutation | -1.05 | 0.348 | |  |  |
| - UPD/ICD versus UBE3A mutation | -0.68 | 0.586 | |  |  |
| **Gender** | 0.4 | 0.604 | |  |  |
| **Epilepsy** | 1.7 | 0.138 | |  |  |

Abbreviations: ICD = Imprinting Center Defect; UPD = Uniparental Paternal Disomy.
